# Mechanistic Insights into Side Effects of Troglitazone and Rosiglitazone Using a Novel Inverse Molecular Docking Protocol

**DOI:** 10.3390/pharmaceutics13030315

**Published:** 2021-02-28

**Authors:** Katarina Kores, Janez Konc, Urban Bren

**Affiliations:** 1 Laboratory of Physical Chemistry and Chemical Thermodynamics, Faculty for Chemistry and Chemical Technology, University of Maribor, Smetanova 17, SI-2000 Maribor, Slovenia; katarina.kores@um.si (K.K.); janez.konc@ki.si (J.K.); 2 Laboratory for Molecular Modeling, Theory Department, National Institute of Chemistry, Hajdrihova 19, SI-1000 Ljubljana, Slovenia; 3 Department of Applied Natural Sciences, Faculty of Mathematics, Natural Sciences and Information Technologies, University of Primorska, Glagoljaška 8, SI-6000 Koper, Slovenia

**Keywords:** thiazolidinediones, rosiglitazone, troglitazone, inverse molecular docking, side effects, CANDOCK

## Abstract

Thiazolidinediones form drugs that treat insulin resistance in type 2 diabetes mellitus. Troglitazone represents the first drug from this family, which was removed from use by the FDA due to its hepatotoxicity. As an alternative, rosiglitazone was developed, but it was under the careful watch of FDA for a long time due to suspicion, that it causes cardiovascular diseases, such as heart failure and stroke. We applied a novel inverse molecular docking protocol to discern the potential protein targets of both drugs. Troglitazone and rosiglitazone were docked into predicted binding sites of >67,000 protein structures from the Protein Data Bank and examined. Several new potential protein targets with successfully docked troglitazone and rosiglitazone were identified. The focus was devoted to human proteins so that existing or new potential side effects could be explained or proposed. Certain targets of troglitazone such as 3-oxo-5-beta-steroid 4-dehydrogenase, neutrophil collagenase, stromelysin-1, and VLCAD were pinpointed, which could explain its hepatoxicity, with additional ones indicating that its application could lead to the treatment/development of cancer. Results for rosiglitazone discerned its interaction with members of the matrix metalloproteinase family, which could lead to cancer and neurodegenerative disorders. The concerning cardiovascular side effects of rosiglitazone could also be explained. We firmly believe that our results deepen the mechanistic understanding of the side effects of both drugs, and potentially with further development and research maybe even help to minimize them. On the other hand, the novel inverse molecular docking protocol on the other hand carries the potential to develop into a standard tool to predict possible cross-interactions of drug candidates potentially leading to adverse side effects.

## 1. Introduction

Thiazolidinediones or glitazones (TZDs) represent established drugs that treat insulin resistance in type 2 diabetes mellitus [1,2], which is a chronic metabolic disorder caused by defects in insulin secretion and insulin action [3]. People with type 2 diabetes mellitus also experience a higher risk for developing cardiovascular diseases [4]. TZDs activate peroxisome proliferator-activated receptor gamma (PPARγ), which is a nuclear receptor that regulates the expression of several genes connected with metabolism. PPARγ represents one of the three PPARs, which are nuclear receptors that regulate lipid metabolism and glucose homeostasis, the latter having control over adipocyte differentiation, lipid storage, and insulin sensitization. The primary effects of TZDs through the activation of PPARγ are seen in adipose tissues (body fat) by reducing triglycerides and increasing HDL cholesterol [1,2,5,6]. Initially, there were a few TZD drugs developed, but only three have been approved by the FDA [2]. To this day, only rosiglitazone (Avandia, Avandaryl, and Avandamet) and pioglitazone (Actos) remain in use. In this study, we focused on two TZDs, troglitazone (Rezulin), which represents the first glitazone-type drug approved by the FDA, but is no longer in use due to its critical side effects, and rosiglitazone, which has been under the watchful eye of the FDA for some time due to suspicion of the potential development of cardiovascular diseases [7].

**Figure 1:**
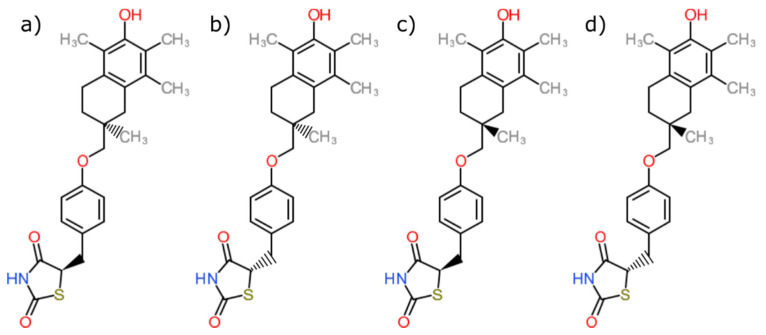
Chemical structures of troglitazone stereoisomers (**a**–**d**). The molecule possesses two chiral centers.

Troglitazone (TGZ) represents the first glitazone antidiabetic agent approved for clinical use. However, due to its hepatotoxicity, Rezulin was withdrawn from the market after a few years [8,9]. TGZ contains a chroman ring of the vitamin E moiety (Figure 1), which could provide TGZ with effective antioxidative properties, but in contrast, it has the potential to undergo metabolic activation, in which several reactive intermediates are produced. These intermediates are toxicologically active [9,10]. Overall, there are multiple potential mechanisms of TGZ hepatotoxicity [8,11] proposed, but to this day, there is no scientific evidence, regarding which is the correct one. TGZ remains a widely researched drug not only in connection with diabetes but also cancer [12,13].

**Figure 2:**
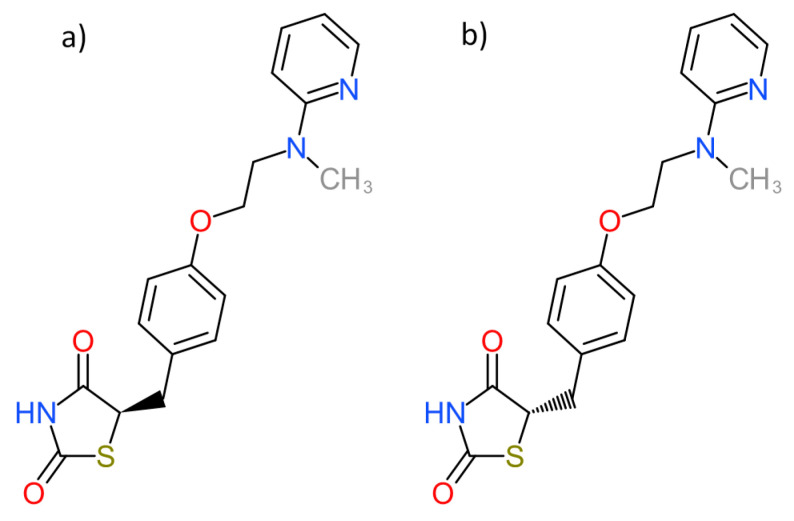
Chemical structures of rosiglitazone enantiomers (**a**,**b**). The molecule possesses one chiral center and is present as a racemate in medicinal applications.

Rosiglitazone (RSG) was designed as an alternative to TGZ since there was no evident connection with hepatotoxicity (Figure 2) [14]. Later, the concern about possible cardiovascular risks was exposed [15,16], and the FDA issued various restrictions on the prescription and application of RSG-containing medicines [17]. After a few years, the FDA lifted the restrictions, as the data collected showed no significant increase in the risk of cardiovascular diseases compared to other drugs [18,19]. The main role of rosiglitazone is to lower insulin resistance, but it also decreases plasma levels of LDL cholesterol, increases levels of HDL cholesterol, creates small changes in triglyceride levels, and decreases blood pressure. These RSG effects could even reduce the increased risk of cardiovascular diseases in patients with type 2 diabetes mellitus [20,21].

Inverse molecular docking represents a new concept in drug discovery, which was now used to identify new potential targets of two thiazolidinediones, RSG and TGZ. Our main goal was to relate these targets with diseases and to use these links to explain the molecular mechanisms of known side effects and, consequently, to anticipate yet unknown, but potential ones. The application of this protocol for identifying possible side effects of drugs in the initial steps of the drug design process has been already reported [22,23,24] and could increase the success rate of drug development as well as its safety [25]. We used an inverse molecular docking protocol coupled with the CANDOCK algorithm [26] and a database of predicted binding sites from the entire Protein Data Bank (PDB) [27] with the ProBiS web server [28]. The novelty of our computational protocol lies in defining the binding sites for a large fraction of the proteins in the PDB and using them for inverse molecular docking [28,29,30]. To the best of our knowledge, this has not been achieved to such an extent ever before. The docking algorithm itself [26] is also new and takes into account aspects such as protein flexibility and protein-ligand scoring using knowledge-based generalized statistical potential functions.

## 2. Materials and Methods

### 2.1. Inverse Molecular Docking

Our goal was to provide mechanistic insights into potential side effects of RSG and TGZ using the CANDOCK inverse molecular docking on more than 67,000 protein structures from various organisms including more than 15,000 human protein structures derived from the PDB. The binding sites for small molecules on the proteins were identified and prepared for inverse molecular docking using the method described in [31] and successfully used for the discovery of new potential targets of the polyphenol curcumin [32]. Focusing the docking search space on the binding sites significantly reduced the time and complexity of the inverse molecular docking. Here, we provide a brief description of the steps involved in preparing this database [31,32].

**Step 1:** For each protein chain (PDB/Chain identifier), the presumed biological structural form was constructed using the data in the header of the corresponding PDB file. Next, the *co*-crystallized ligands of the protein chain identified by HETATM records with >7 heavy atoms, with at least one atom less than 3 Å away from the respective protein chain, were considered. Binding sites were determined from each extracted ligand, i.e., protein atoms < 5 Å away from all ligand atoms. Finally, binding site surface files were generated that served as an input to the ProBiS algorithm in a subsequent step.

**Step 2:** The ~310,000 protein chains in the PDB were clustered using a sequence identify cutoff at 100%, resulting in 70,000 protein chain clusters. The binding sites obtained in Step 1 were assigned to the corresponding sequence clusters. Each binding site was assigned to a cluster containing the protein (identified by PDB/Chain identifier) from which it was extracted. Water molecules and cofactors within the binding pocket were removed prior to inverse molecular docking simulation.

**Step 3:** Each pair of binding sites was compared within each cluster with 100% sequence identity in an “all versus all” fashion using the ProBiS algorithm [28,29]. The ProBiS algorithm allows for the detection of structurally similar protein binding sites as well as for the local pairwise alignment of X-ray or NMR determined protein structures from the PDB [29].

**Step 4:** Then, the binding sites within each cluster with 100% sequence identity were then clustered. In this second clustering, the similar binding site pairs (with z-score ≥ 2.0) were assigned to the same cluster. This clustering of binding sites within the existing sequence identity clusters ensures that if a protein has two or more distinct binding sites, each can be assigned to a distinct binding site cluster.

**Step 5:** The representative binding site surfaces (approximately 35,000 surfaces) were selected along with the co-crystallized ligands that were transferred to the representative binding sites.

**Step 6:** Then, the pre-calculated database of binding sites and ligands was then processed for each existing protein structural chain in the PDB (~310,000) by comparing that particular chain to the binding site surfaces using the ProBiS algorithm (Step 5). The identified structural similarities allowed for the prediction of binding sites for protein chains (even non-representative ones) for which binding sites had not yet been identified. Binding sites were defined as the union of centroids, i.e., the total space occupied by multiple spheres following the contours of the binding site.

**Step 7:** Then, we then filtered the obtained database of binding sites of small molecule ligands (consisting of 67,053 binding sites from different organisms) for the human proteins, resulting in a total of 15,482 binding sites of small molecule ligands to human proteins, which were subsequently applied as input for the inverse molecular docking using the newly developed CANDOCK program [26].

The CANDOCK algorithm applies a hierarchical approach to reconstruct small molecules from the atomic grid using graph theory and generalized statistical potential functions. Therefore, the docking scores represent approximations of the relative binding free energies and have arbitrary units. It docks fragments of small molecules into binding sites of investigated proteins using knowledge-based scoring methods. CANDOCK finds the best-docked poses of fragments and applies a fast-maximum clique algorithm [33] to link them together. In reconstructing the molecule, the algorithm uses iterative dynamics for a better placement of the ligand in the binding pocket. A second step represents the minimization of the docking score, using different methods that model the flexibility of the ligand and amino acid residues in the binding site [26,30,32,34].

RSG and TGZ structures, prepared with chemical editor Avogadro [35], served as input to the algorithm. These structures were subsequently optimized with Gaussian 16 [36], an electronic structure program, in conjunction with the *Hartree-Fock* method and *6-31G* basis set. Then, a variety of multi-organism proteins along with the coordinates of their binding sites, defined as centroids, was used as targets. In each target, we docked all enantiomers of RSG and TGZ and took into consideration poses with the lowest predicted knowledge-based score. 

**Figure 3:**
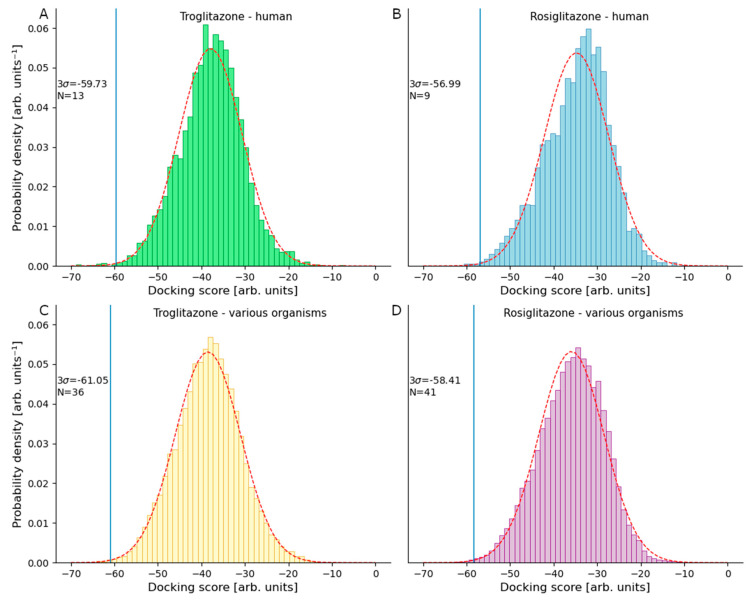
Normal distribution fitting graphs of inverse docking scores for (**A**) troglitazone docked into human targets, (**B**) rosiglitazone docked into human targets, (**C**) troglitazone docked into targets from various organisms and (**D**) rosiglitazone docked into targets from various organisms. 99.7% confidence interval is marked as 3σ and the number of proteins that fit the selected criteria is denoted with N.

TGZ and RSG were successfully docked to more than 21,000 proteins from various organisms, of which slightly less than 5000 formed human protein targets. From the calculated conformations of all stereoisomers of RSG and TGZ we prepared two ranked lists for each molecule, one of multi-organism targets and the other of human targets. We selected the highest-ranking proteins from all four lists based on the 99.7% confidence interval criteria (Figure 3). As potential targets, we explicitly considered all proteins with docking scores below the selected confidence interval.

### 2.2. Binding Site Comparisons Using ProBiS Web Server

In the PDB, one finds a few known targets of troglitazone and rosiglitazone. At this stage, we searched for protein structures that have structurally similar binding sites to those where RSG or TGZ are already co-crystallized. To achieve this, we utilized the ProBiS web server [29] (http://probis.cmm.ki.si/, accessed on 28 January 2021).

We used all PDB protein structures containing TGZ or RSG as queries for the ProBiS web server. The structural similarity was determined with Z-score, the number of standard deviations from the mean data point. Z-score is used for the calculation of similarity between the selected and the query protein compared with a randomly selected protein. The higher the Z-score, the more similar the two proteins. We considered similarities at distances of 7.0 Å around the bound TGZ or RSG. We took only human protein structures with the highest similarity to each of the already known targets into consideration and selected them according to Z-score (≥2.00).

### 2.3. Method Validation

To validate our method, we used a redocking procedure, receiver operating characteristics (ROC) curves [37], enrichment curves [38], and predictiveness curves (PC) [39]. We obtained 12 crystal protein structures from the PDB, which have a co-crystallized ligand TGZ or RSG bound in their binding sites. We removed the co-crystallized ligands but left the potential cofactors. Then we redocked TGZ or RSG to their respective protein structures. The validity of the method was determined with root-mean square deviation (RMSD) of atomic positions between co-crystallized and docked ligands [34]. Figures of TGZ and RSG in in their respective binding sites were prepared using UCSF Chimera [40]. The ROC metric plot shows a correlation between the true positive fraction (TPF) on the y-axis and the false positive fraction (FPF) on the x-axis. In our case, TPF represents experimentally confirmed protein targets from the ChEMBL database [41] for TGZ or RSG with the corresponding PDB entry, while FPF represents protein targets that successfully bound TGZ or RSG. The area under the ROC curve (ROC AUC) is used as a practical method to measure the overall performance. The larger the ROC AUC, the more effective the inverse docking is in distinguishing active from inactive compounds [37]. The enrichment curve represents the early detection quantification of target proteins from TPF [38]. PC also provides the early detection quantification of target proteins from TPF. In addition, the PC can be used to define a score threshold for potential targets from the inverse docking to be tested experimentally [39]. For quantifying early detection, the enrichment factor of 1% of screened compounds (EF) [42], Boltzmann-enhanced discrimination of ROC (BEDROC) [38], and the robust initial enhancement (RIE) [42] are used. Applying PC, the standardized total gain (TG) can be calculated. The TG summarizes the contribution of the inverse docking protocol scores in explaining the activity of compounds over the entire protein dataset [39]. We used a web-based interactive application Screening explorer [43] that covers all aspects of the presented analysis.

## 3. Results

### 3.1. Targets of Troglitazone in the Human Proteome

For the analysis of potential disease treatments and potential side effects of TGZ, we selected the 13 highest-ranking human target proteins according to the 99.7% confidence interval. In our cut off, one could find some protein targets that were represented by two (PDB IDs: 3uzxA, 5fyyA) or even three (PDB ID: 4gs4A) PDB entries and had more than 95% sequence similarity. The only differences between these similar proteins were SNPs as well as initial and ending tails. Since our investigation was focused on finding potential protein targets regardless of small details, we took into consideration only the highest-ranking representative for each match. Table 1 lists the human proteins in which the predicted docking score of TGZ was the most favorable.

The enzyme 3-oxo-5-beta-steroid 4-dehydrogenase controls the expression and activity of cytochrome P450 family. With its inhibition, the expression of P450s is reduced, which directly affects drug and toxin metabolisms in the human liver [61,62]. Lysine-specific demethylase 5B forms a potential tumor suppressor, and with its inhibition, this function could be lost, potentially leading to the development of cancer [46]. Aurora kinase A is known for its overexpression in a variety of cancer types, where its inhibition represents a potential treatment. However, in healthy cells, its inhibition could interfere with normal mitosis [47,48]. With binding of TGZ into alpha-tubulin *N*-acetyltransferase 1, the dysregulation of protein function could occur leading to abnormal levels of tubulin acetylation, which have been already linked to a variety of neurological disorders, heart diseases, and cancer [50]. The inhibition of neutrophil collagenase could successfully treat degenerative diseases with a slow matrix degradation rate. However, its inhibition in healthy cells could generate disorders of collagen degradation, causing a variety of diseases, such as liver cirrhosis. One could expect an analogous behavior for another protein from our set, Stromelysin-1 [52,58,63]. The binding of TGZ into *N*-alpha-acetyl transferase 60 could cause abnormal chromosome segregation, which triggers the development of cancer [64]. As d-amino-acid oxidase plays a detoxification role in the liver, the toxicity of the accumulated d-amino-acids would increase in the case of its inhibition [56]. The VLCAD protein is implicated in the oxidation of fatty acids, which are used to synthesize ketone bodies in the liver, the so-called fuel for the brain and muscle. Inhibition of VLCAD could lead to liver damage, other serious problems, and even death [60]. Its inhibition in the neonate or toddler groups could cause a variety of diseases, ranging from muscle weakness, over developmental regression and behavioral disorders, until attention deficit disorder [65].

Since molecular docking was performed separately for all stereoisomers of TGZ, the highest ranked human targets according to the 99.7% confidence interval of each isomer are presented in the Supplementary Material, Tables S1–S4. As can be seen from these tables, the diversity of protein targets is isomer specific, but there are a few common targets, such as 3-oxo-5-beta-steroid 4-dehydrogenase, lysine-specific demethylase 5B, and aurora kinase A. A table of the highest ranked protein targets of TGZ from different organisms is also shown in the Supplementary Material (Table S7).

### 3.2. Targets of Rosiglitazone in the Human Proteome

Nine of the highest-ranking human target proteins of RSG were selected (based on the 99.7% confidence interval), and potential diseases treatments and potential side effects were analyzed. Table 2 lists the nine human protein targets in which the predicted docking score of RSG was the most favorable. 

RSG possesses a known connection only with the first protein target from our list, matrix metalloproteinase 9 [90], which is essential for collagen degradation. Disorders of collagen degradation cause a variety of diseases, such as arthritis and liver cirrhosis [63]. One could expect similar side effects for collagenase 3 from our list. UDP-glucuronic acid decarboxylase 1 catalyzes the formation of UDP-xylose, and its inhibition could reduce concentrations of xylose and consequently increase expressions of the proteins involved in the formation of hyaluronan, which could led to the development of cancer [91]. RSG could also be a new inhibitor of KMT5A protein, whose inhibition could lead to the dysregulation of protein methylation, which is directly connected with neurodegenerative diseases and cancer [73]. By a selective inhibition of DNA (cytosine-5)-methyltransferase 3A (DNMT3A), one could regulate DNA methylation. However, in the case of absence or errors in DNA methylation, several disorders occur, such as certain cancer types, defects in embryonic and brain development, or neurological diseases [77]. Estradiol is known for its anti-inflammatory, neuroprotective, and neuroregenerative actions in neurological disorders such as Alzheimer’s disease, Parkinson’s disease, multiple sclerosis, and cerebrovascular stroke [92]. The inhibition of estradiol 17-beta-dehydrogenase 1 by RSG could lead to a reduced production of estradiol thereby decreasing its anti-inflammatory actions. The dysregulation of guanine nucleotide-binding protein by RSG could interfere with the production of cAMP. Decreased cAMP concentrations could lead to hepatocellular carcinoma while sustained elevation results in cell death [85,93]. Inhibition of UDP-glucose 4-epimerase could result in a reduced concentration of d-galactose, which is important for the maintenance of cell surface polysaccharide patterns [94], and EEF1A lysine methyltransferase 4 inhibition could cause alterations in the translation of several mRNAs.

Since molecular docking was performed separately for both stereoisomers of RSG, the highest ranked human targets according to the 99.7% confidence interval of each isomer are shown in Supplementary Material, Tables S5 and S6. As can be observed from these tables, most of the protein targets differ between isomers. A table of the highest ranked protein targets of RSG from different organisms is also shown in the Supplementary Material (Table S8).

### 3.3. Binding Site Comparison Using ProBiS Web Server

#### 3.3.1. Troglitazone

In the PDB, one finds a single human protein structure with co-crystallized troglitazone, Cytochrome P450 2C8 (PDB ID: 2vn0, chain A). We compared the binding site of troglitazone from this known structure against the entire PDB using the ProBiS web server and obtained a list of human proteins with structurally similar (Z-score larger than 2) binding sites to the crystallized target (Table 3).

All proteins found with the ProBiS web server belong to the same Cytochrome P450 family. All possess very similar 3D structures, but different sequences (from 30–70% sequence similarity with the notable exception of lanosterol 14-alpha demethylase: 3ld6A). 

#### 3.3.2. Rosiglitazone

In the PDB, one finds 11 human protein structures with co-crystallized rosiglitazone. All PDB entries represent the same peroxisome proliferator-activated receptor gamma (PPARγ) protein. PPARγ is a member of the hormone receptor family, but its role in the heart is not well understood, besides the fact that its overexpression in the heart tissue can lead to cardiac dysfunction [95]. We compared these structures to the entire PDB, using the ProBiS web server and obtained the list of human proteins with structurally similar (Z-score larger than 2) binding sites to query proteins with co-crystallized rosiglitazone (Table 4).

All query structures with bound rosiglitazone are within 95–100% sequence similarity, with the notable exception of 3dzyD, which has around 50% sequence similarity with the others because of its longer chain. However, the binding site possesses an almost identical sequence in all query proteins, 3dzy included.

As one can see from Table 4, the identified proteins are virtually the same for all known binders, with the addition of two notable exceptions, Oxysterols receptor LXR-alpha (PDB ID: 3ipq) and MIT domain-containing protein 1 (PDB ID: 4a5x). 

#### 3.3.3. Method Validation

**Figure 4:**
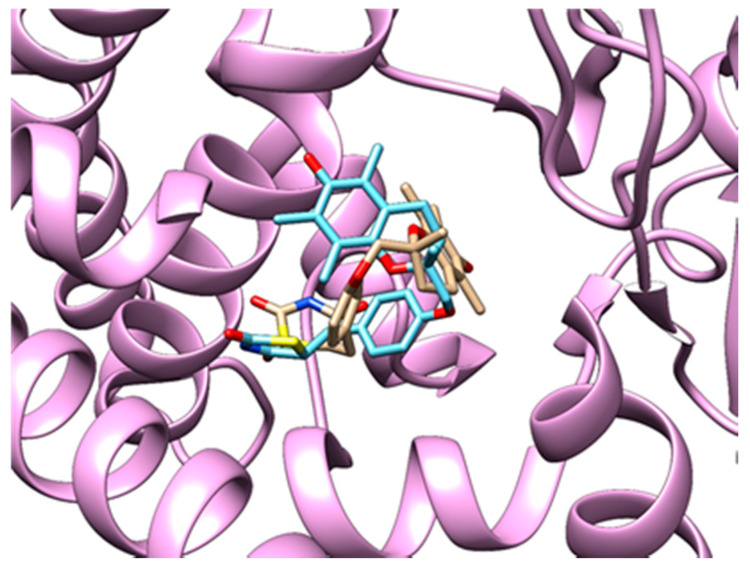
Native (blue) and redocked (brown) poses of troglitazone in the cytochrome P450 2C8 protein (light purple). RMSD: 4.51 Å.

**Figure 5:**
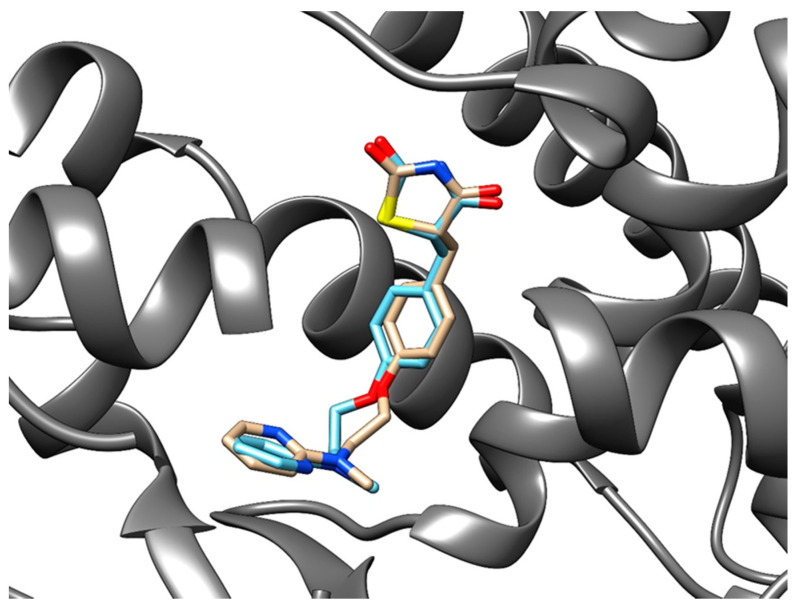
Native (blue) and redocked (brown) poses of rosiglitazone in the peroxisome proliferator-activated receptor gamma protein (gray). RMSD: 0.85 Å.

**Redocking procedure.** We obtained a structure with co-crystallized troglitazone from PDB (PDB ID: 2vn0, chain A), which we used in the redocking procedure. We took into the consideration three highest-scoring poses for all four stereoisomers of troglitazone (Table 5). We also obtained 11 human protein structures with co-crystallized rosiglitazone. For each structure, we considered three highest-scoring poses for each enantiomer of rosiglitazone (Table 6). For each pose, we calculated the RMSD between the docked and the native positions from the PDB. We reviewed all considered poses and selected the best ones (denoted in green) based on a combination of RMSD and correct chirality according to the native poses of co-crystallized rosiglitazone or troglitazone. RMSD values are a little higher than expected [96], but that could well be a direct consequence of molecular size and its chirality. In Figure 4 comparison of the poses of native (blue) and docked (brown) troglitazone in cytochrome P450 2C8 is depicted (PDB ID: 2vn0, chain A). Figure 5 presents a comparison of the native (blue) and docked (brown) rosiglitazone poses in peroxisome proliferator-activated receptor gamma protein (PDB ID: 2prg, chain A). 

**Figure 6:**
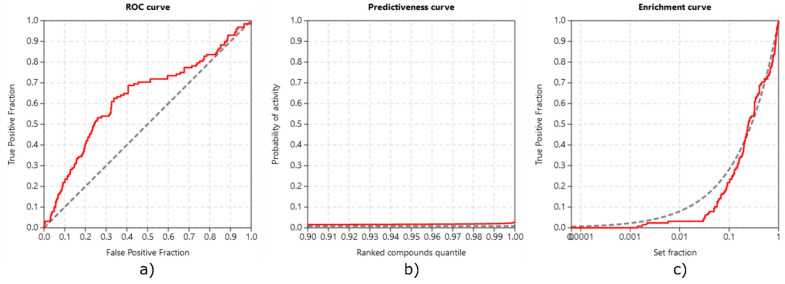
Validations of inverse molecular docking protocol of troglitazone against all human protein targets from the PDB: (**a**) the receiver operating characteristics (ROC) curve; (**b**) the predictiveness curve; and (**c**) the enrichment curve.

**Figure 7:**
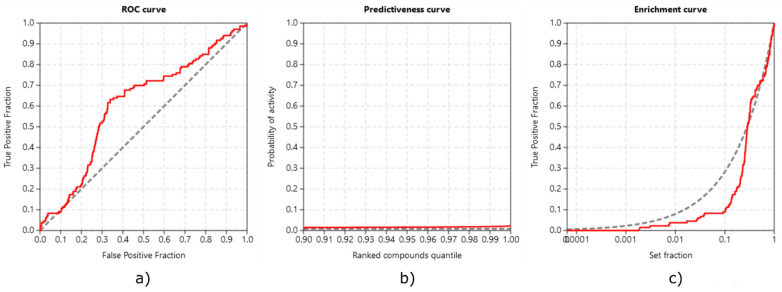
Validations of inverse molecular docking protocol of rosiglitazone against all human protein targets from the Protein Date Bank (PDB): (**a**) the receiver operating characteristics (ROC) curve; (**b**) the predictiveness curve; and (**c**) the enrichment curve.

**Validation with ROC, EF, and PC.** We performed inverse molecular docking using the CANDOCK algorithm on a set of 15,482 human proteins, including 128 confirmed target proteins of TGZ (and 133 of RSG) whose measured IC_50_ values for binding TGZ (or RSG) were <10 µM [41]. The inverse docking was successfully performed on 69 (TGZ) and 74 (RSG) of those confirmed targets. The ability of CANDOCK to distinguish the confirmed protein targets of TGZ and RSG from non-target proteins was assessed using established metrics, which are shown in Figure 6 (TGZ) and in Figure 7 (RSG).

Our protocol was successful in discriminating between true targets of TGZ (ROC AUC of 0.631) and RSG (ROC AUC of 0.606). Early detection of protein targets with BEDROC of 0.110 and 0.078, RIE of 2.030 and 1.431, and EF1% of 1.2095 and 1.1641, for TGZ and RSG, respectively, was satisfactory. The protocol produced score variations in the detection of true target proteins (TG 0.236 and 0.189 for TGZ and RSG, respectively), which, in combination with the ROC AUC above 0.6, demonstrates that the protocol is expected to provide a satisfactory agreement with experiments [39].

Our inverse docking protocol was previously extensively validated by Fine and Konc et al. [26], Furlan et al. [32], and Kores et al. [34].

## 4. Discussion

Predicting potential side effects of new drugs represents one of the key stages in drug development [22]. This is also important for existing drugs that have been on the market for a long time. Here, it is important to explain the molecular mechanisms behind why the already known side effects occur, and thus potentially predict new ones that may still occur or have not yet been associated with this drug. The mechanistic understanding may even afford directions on how to alleviate these side effects. 

TGZ and RSG are known agonists of human PPARγ. In our set of targets where both drugs were successfully docked, there are four structures of the said protein (PDB IDs: 3dzuD, 4r6sA, 4oj4A, 3u9qA). The docking scores for TGZ ranged from −43 to −58, while the scores for RSG ranged from −37 to −45. These results indicate that the docking scores of TGZ and RSG docked to the PPARγ are higher and thus less favorable than our proposed targets within the 99.7% confidence interval, implying that these new potential targets may indeed contribute to the understanding of the side effects of both drugs. In addition, the redocking scores of RSG are similar to those from inverse docking for human PPARγ. 

Troglitazone was withdrawn from use due to potential liver defects [8]. In our computational study, troglitazone indeed successfully docked into certain protein structures, which have direct or indirect impacts on the liver [52,60,61,63]. The highest-ranking targets included 3-oxo-5-beta-steroid 4-dehydrogenase [44], neutrophil collagenase [51], stromelysin-1 [57], and VLCAD [59], which could all be involved in the development of hepatotoxicity. Troglitazone also successfully docked into certain protein structures such as lysine-specific demethylase 5B [45], aurora kinase A [47], and alpha-tubulin *N*-acetyltransferase 1 [50], which are connected to cancer. As the latest findings show, troglitazone could indeed be used to treat various cancer types [97,98], but our results indicate that troglitazone could also trigger carcinogenesis [46,64]. None of our highest-ranking targets has known connections with troglitazone. Binding site comparison using ProBiS webserver returned targets with similar binding sites, which all belonged to the same cytochrome P450 family. Enzymes from this family are very important in drug metabolism, and in the case of troglitazone, because of its higher dosages, the covalent bonding to different CYP enzymes could indeed result in an increased risk of hepatotoxicity [99]. 

Rosiglitazone became accepted as an alternative to troglitazone, since there were no indications that it causes hepatotoxicity [14]. Rosiglitazone is known to have an experimental connection with the matrix metalloproteinase family (MMPs), and two of its members are indeed among our top targets. MMPs play an important role in tissue remodeling and repair. So, any irregularity could lead to various diseases, such as cancer, neurological disorders, or cardiovascular diseases [63,69,79]. This could also represent the explanation behind the possible cardiovascular risk, which caused multiple restrictions on the application of rosiglitazone-containing drugs [15,17]. Dysregulation or inhibition of certain proteins with rosiglitazone could potentially lead to cancer [77,91,93] and neurodegenerative diseases [73,77,92] as well. Binding site comparison was performed on 11 PDB entries representing the same PPARγ protein, which is known as the main target of glitazones [6]. The results discern the potential binding of rosiglitazone to two additional proteins from the PPAR family. Therefore, we could talk about PPAR co-agonists. PPAR co-agonists were indeed tested in the past with several promising leads, but there is very little known about the possible side effects of these kinds of drugs [6,100].

The main limitation of the inverse docking approach is that not all human proteins are available in the PDB yet, as this is the requirement of our protocol. However, proteome-wide structural coverage is already available for most pharmaceutically relevant protein classes, especially for humans [101]. We strongly believe that the application of our approach can provide important results to improve not only the drug design and discovery but also the management of potential side effects in most known human diseases and disorders.

## 5. Conclusions

In this paper, we focused on two drugs from the thiazolidinedione family, troglitazone, which was the first of its type but was withdrawn from use due to its severe side effects, and rosiglitazone, which was under FDA evaluation for a long time as a potential trigger of cardiovascular diseases [7]. We used the in-house developed inverse docking algorithm CANDOCK [26]. Structures of both drugs were prepared with Avogadro [35] and optimized with Gaussian [36], and the database of protein structures was prepared from the online Protein Data Bank [102]. In addition, a binding site comparison with ProBiS web server [29] was performed. 

Our results successfully explained the side effects of troglitazone and rosiglitazone, and we also predicted additional ones, that could occur after the long-term application of these two drugs. With further development of computational methods and in conjunction with experimental research, these results can be further analyzed to better explain the side effects of both drugs and to help mitigate or even eliminate them. A detailed study of the individual isomers on the protein targets could also help to develop TGZ and RSG drugs with only those stereoisomers that have a beneficial effect on humans and do not show negative side effects. On the other hand, the novel inverse molecular docking protocol could be generally applied to discern possible cross-interactions of drug candidates, potentially leading to adverse side effects.

## Figures and Tables

**Table 1:** Potential human target proteins of troglitazone.

PDB ID with Chain	Docking Score [Arbitrary Units ^1^]	Protein Name	Protein Function and Connection with Diseases	References
3uzxA ^2^	−68.2	3-oxo-5-beta-steroid 4-dehydrogenase	Catalyzes the reduction of the Δ4 double bond of bile acid intermediates and steroid hormones carrying the Δ4-3-one structure in the A/B cis configuration.	[44]
5fyyA ^2^	−68.0	Lysine-specific demethylase 5B	A member of the KDM5 sub-family, acting as a histone 3 lysine 4 trimethyl demethylase, regulating proliferation, stem cell self-renewal, and differentiation. Connected with various forms of cancer.	[45,46]
2wtvA	−66.1	Aurora kinase A	Involved in the regulation of centrosomes and segregation of chromosomes. Associates with the centrosome and the spindle microtubules during mitosis and plays a critical role in several mitotic events. Known connections to various forms of cancer.	[47,48]
4gs4A ^3^	−64.7	Alpha-tubulin *N*-acetyltransferase 1	Acetylates K40 on α-tubulin at the lumenal side of microtubules. Connections with neurological disorders, cancer, heart diseases.	[49,50]
1mncA	−64.0	Neutrophil collagenase	A member of the matrix metalloproteinase family. Degrades fibrillar type I, II, and III collagens. Implicated in several degenerative diseases with a slow matrix degradation rate, such as osteoporosis and Alzheimer’s disease.	[51,52]
5hh1A	−63.5	*N*-alpha-acetyl transferase 60	Specifically catalyzes acetylation of the N-terminal α-amine in a majority of transmembrane proteins, mediates lysine acetylation of free histone H4. Possible connections with different forms of cancer.	[53]
3znnA	−62.7	d-amino-acid oxidase	Catalyzes the oxidative deamination of d-isomers of neutral and polar amino acids. The physiological role in the kidney and liver represents the detoxification of accumulated d-amino acids. Known connection with schizophrenia.	[54,55,56]
1c8tA	−62.3	Stromelysin-1	A member of the matrix metalloproteinase family. It degrades numerous ECM substrates, including collagens III, IV, V, IX, X, and XI, laminins, and elastin. Known connections with fibrosis, neovascularization, and potentially cancer progression.	[57,58]
3b96A	−60.3	Very long-chain specific acyl-CoA dehydrogenase, mitochondrial (VLCAD)	Catalyzes the initial, rate-limiting step of the mitochondrial fatty acid β-oxidation, exerts optimal chain length specificity for fatty acyl-CoAs having 16–24 carbons in length. Connected with hypoketotic hypoglycemia, liver damage, and hypertrophic myocardiopathy.	[59,60]

^1^ Predicted knowledge-based docking scores with arbitrary units reflect relative binding free energies of a ligand to a protein. ^2^ The highest-ranking representative of a protein possessing two PDB entries in our list. ^3^ The highest-ranking representative of a protein possessing three PDB entries in our list.

**Table 2:** Potential human target proteins of rosiglitazone.

PDB ID with Chain	Docking score [Arbitrary Units ^1^]	Protein Name	Protein Function and Connection with Diseases	References
4jijA	−60.5	Matrix metalloproteinase 9	Degrades components of the extracellular matrix, mostly collagens. Known connections with cancer, neurological disorders, and cardiovascular diseases.	[66,67,68,69]
4lk3A	−59.9	UDP-glucuronic acid decarboxylase 1	Catalyzes the formation of UDP-xylose from UDP-glucuronate. No known connections with human diseases.	[70,71]
3f9zA	−59.8	*N*-lysine methyltransferase KMT5A	Monomethylates lysine 20 of histone H4 (H4K20), proliferating cell nuclear antigen (PCNA) and p53. Potential connections with cancer tumorigenesis and neurodegenerative diseases.	[72,73]
4u7pA	−58.8	DNA (cytosine-5)-methyltransferase 3A	Responsible for the de novo methylation of 5-methylcytosine during embryogenesis for the establishment of the somatic methylation pattern, which is crucial for embryonic development. Known connections with hematological malignancies, that is tumors of the hematopoietic and lymphoid tissues, as well as with defects in brain development or neurological defects.	[74,75,76,77]
4fvlA	−58.6	Collagenase 3	One of the matrix metalloproteinases. Plays a key role in tissue remodeling and repair. Known connections with cancer, aneurysm, fibrosis, and other diseases.	[78,79,80]
1fduA	−58.4	Estradiol 17-beta-dehydrogenase 1	Catalyzes the reversible transformation of estrone into biologically active estradiol. Connected with cancer, the biology of reproduction, and neuronal diseases.	[81,82]
5tdhA	−58.0	Guanine nucleotide-binding protein G(i) subunit alpha-1	Transduces intracellular signals from membrane-bound receptors to downstream effector molecules. Cooperates in different biological activities, such as vision or synaptic nerve signal transmissions. Connected with neurological problems, cardiovascular defects, and certain forms of cancer.	[83,84,85]
1i3kA	−57.6	UDP-glucose 4-epimerase	Catalyzes the interconversion of UDP-galactose and UDP-glucose during normal galactose metabolism, as well as interconversion of UDP-N-acetyl galactosamine (UDP-galNAc) and UDP-N-acetylglucosamine (UDP-glcNAc). Loss of this protein results in the spectrum disorder epimerase deficiency galactosemia.	[86,87,88]
2pxxA	−57.6	EEF1A lysine methyltransferase 4	Catalyzes methylations on Lys36 in eukaryotic translation elongation factor 1 alpha. No known connections with human diseases.	[89]

^1^ Predicted knowledge-based docking scores with arbitrary units reflect relative binding free energies of a ligand to a protein.

**Table 3:** Cytochrome P450 2C8 structure with co-crystallized troglitazone as query protein and the identified human proteins with structurally similar binding sites.

Query Protein	Proteins with Similar Binding Sites		
PDB ID with Chain	PDB ID with Chain	Protein	Z-Score
2vn0A	1r9oA	Cytochrome P450 2C9	3.83
	4gqsA	Cytochrome P450 2C19	3.82
	3tbgA	Cytochrome P450 2D6	3.21
	3ibdA	Cytochrome P450 2B6	3.13
	3czhA	Cytochrome P450 2R1	3.11
	4nkyA	Steroid 17-Alpha-Hydroxylase/17,20 Lyase	3.00
	4y8wA	Cytochrome P450 21-Hydroxylase	2.93
	3e6iA	Cytochrome P450 2E1	2.54
	3ld6A	Lanosterol 14-Alpha Demethylase	2.06

**Table 4:** Peroxisome proliferator-activated receptor gamma structures with co-crystallized rosiglitazone as query proteins and the identified human proteins with structurally similar binding sites.

Query Proteins	Proteins with Similar Binding Sites		
PDB ID with Chain	PDB ID with Chain	Protein	Z-Score
1fm6DX	3vi8A	Peroxisome Proliferator-Activated Receptor Alpha	3.84
	3tkmA	Peroxisome Proliferator-Activated Receptor Delta	3.77
	3ipqA	Oxysterols Receptor LXR-Alpha	2.04
1zgyA	3vi8A	Peroxisome Proliferator-Activated Receptor Alpha	3.78
	3tkmA	Peroxisome Proliferator-Activated Receptor Delta	3.66
2prgAB	3vi8A	Peroxisome Proliferator-Activated Receptor Alpha	3.60
	3tkmA	Peroxisome Proliferator-Activated Receptor Delta	3.59
3cs8A	3tkmA	Peroxisome Proliferator-Activated Receptor Delta	3.30
	3vi8A	Peroxisome Proliferator-Activated Receptor Alpha	2.98
3dzyD	3vi8A	Peroxisome Proliferator-Activated Receptor Alpha	3.25
	3tkmA	Peroxisome Proliferator-Activated Receptor Delta	3.05
4emaA	3vi8A	Peroxisome Proliferator-Activated Receptor Alpha	3.73
	3tkmA	Peroxisome Proliferator-Activated Receptor Delta	3.14
4o8fAB	3tkmA	Peroxisome Proliferator-Activated Receptor Delta	3.63
	3vi8A	Peroxisome Proliferator-Activated Receptor Alpha	3.45
4xldA	3vi8A	Peroxisome Proliferator-Activated Receptor Alpha	3.85
	3tkmA	Peroxisome Proliferator-Activated Receptor Delta	3.44
5ji0D	3tkmA	Peroxisome Proliferator-Activated Receptor Delta	3.94
	3vi8A	Peroxisome Proliferator-Activated Receptor Alpha	3.86
5ycpA	3vi8A	Peroxisome Proliferator-Activated Receptor Alpha	3.15
	3tkmA	Peroxisome Proliferator-Activated Receptor Delta	2.73
	4a5xA	MIT Domain-Containing Protein 1	2.04
6md4A	3tkmA	Peroxisome Proliferator-Activated Receptor Delta	3.76
	3vi8A	Peroxisome Proliferator-Activated Receptor Alpha	3.36

**Table 5:** Results of the redocking procedure for troglitazone.

PDB ID with Chain (Crystal Structure)	Stereoisomer (Figure 1)	Docking Score [Arbitrary Units]	RMSD [Å]
2vn0A	a	−24.77	4.51
	b	−14.1	5.45
	c	−26.5	5.84
	d	−25.1	6.12

**Table 6:** Results of the redocking procedure for rosiglitazone.

PDB ID with Chain (Crystal Structure)	Stereoisomer (Figure 2)	Docking Score [Arbitrary Units]	RMSD [Å]
1fm6D	a	−30.6	2.15
	b	−32.1	2.25
1zgyA	a	−32.9	2.26
	b	−38.2	2.47
2prgA	a	−37.1	0.85
	b	−46.2	2.70
3cs8A	a	−29.0	2.89
	b	−28.2	8.07
3dzyD	a	−41.4	2.22
	b	−32.0	1.09
4emaA	a	−43.6	1.96
	b	−21.0	3.20
4o8fA	a	−32.2	1.12
	b	−23.8	2.03
4xldA	a	−39.3	1.78
	b	−35.8	2.62
5ji0D	a	−37.2	1.44
	b	−39.7	2.14
5ycpA	a	−34.3	1.76
	b	−44.9	1.90
6md4A	a	−31.8	2.06
	b	−37.6	2.62

## Data Availability

All data generated or analysed during this study are included in this published article.

## Supplementary Materials

The following are available online at https://www.mdpi.com/1999-4923/13/3/315/s1, Table S1: Potential human targets of troglitazone stereoisomer a (Figure 1), Table S2: Potential human targets of troglitazone - stereoisomer b (Figure 1), Table S3: Potential human targets of troglitazone stereoisomer c (Figure 1), Table S4: Potential human targets of troglitazone stereoisomer d (Figure 1), Table S5: Potential human targets of rosiglitazone stereoisomer a (Figure 2), Table S6: Potential human targets of rosiglitazone stereoisomer b (Figure 2), Table S7: Potential target proteins of troglitazone in various organisms, Table S8: Potential target proteins of rosiglitazone in various organisms.
