# Supplementary material for: Mechanistic Insights into Side Effects of Troglitazone and Rosiglitazone Using a Novel Inverse Molecular Docking Protocol
Source: Pharmaceutics. 2021 Feb 28;13(3):315. doi: 10.3390/pharmaceutics13030315 (PMC7997210; doi:10.3390/pharmaceutics13030315)
Supplement: Supplementary file 1 [file pharmaceutics-13-00315-s001.pdf]

pharmaceutics

Supplementary Material: Mechanistic Insights into Side Effects of Troglitazone and Rosiglitazone Using a Novel Inverse Molecular Docking Protocol

Katarina Kores, Janez Konc and Urban Bren

Table S1. Potential human targets of troglitazone - stereoisomer a (Figure 1).

PDB ID with chain	Docking score [Arbitrary Units[1]]	Protein name
5fyyA	−72.8685	Lysine-specific demethylase 5B
1mncA	−69.5856	Neutrophil collagenase
3uzxA	−68.7357	3-oxo-5-beta-steroid 4-dehydrogenase
2wtvA	−67.686	Aurora kinase A
3oiwA	−66.7198	GTPase HRas
4gs4A	−65.4331	Alpha-tubulin N-acetyltransferase 1
5fyzA	−64.8656	Lysine-specific demethylase 5B
5hh1A	−64.6355	N-alpha-acetyltransferase 60
1xu9A	−64.6251	Corticosteroid 11-beta-dehydrogenase isozyme 1
3tsoA	−64.4635	Ras-related protein Rab-25
3znnA	−64.4205	D-amino-acid oxidase
2chmA	−64.2897	cGMP-specific 3',5'-cyclic phosphodiesterase
1c8tA	−63.7248	Stromelysin-1
2ob0A	−63.4888	N-alpha-acetyltransferase 50
3buvA	−63.4469	3-oxo-5-beta-steroid 4-dehydrogenase
3o8uA	−63.3234	Mitogen-activated protein kinase 14
3qe2A	−63.0633	NADPH--cytochrome P450 reductase

[1] Predicted knowledge-based docking scores with arbitrary units reflect relative binding free energies of a ligand to a protein.

Table S2. Potential human targets of troglitazone - stereoisomer b (Figure 1).

PDB ID with Chain	Docking Score [Arbitrary Units[1]]	Protein Name
5fyzA	−70.2886	Lysine-specific demethylase 5B

5fyyA	−68.4725	Lysine-specific demethylase 5B
3uzxA	−67.4028	3-oxo-5-beta-steroid 4-dehydrogenase
3b96A	−66.2273	Very long-chain specific acyl-CoA dehydrogenase, mitochondrial
2ac3A	−65.627	MAP kinase-interacting serine/threonine-protein kinase 2
3tsoA	−65.3645	Ras-related protein Rab-25
5hh1A	−65.3143	N-alpha-acetyltransferase 60
3buvA	−65.2469	3-oxo-5-beta-steroid 4-dehydrogenase
2ipjA	−64.5561	Aldo-keto reductase family 1 member C2
1n6oA	−63.9221	Ras-related protein Rab-5A
2wtvA	−63.437	Aurora kinase A
4kzjB	−63.1215	Nuclear receptor subfamily 4 group A member 1
1xg5A	−62.7147	Dehydrogenase/reductase SDR family member 11

[1] Predicted knowledge-based docking scores with arbitrary units reflect relative binding free energies of a ligand to a protein.

Table S3. Potential human targets of troglitazone - stereoisomer c (Figure 1).

PDB ID with Chain	Docking Score [Arbitrary Units[1]]	Protein Name
3uzxA	−69.5925	3-oxo-5-beta-steroid 4-dehydrogenase
2wtvA	−68.8161	Aurora kinase A
5fyyA	−68.2795	Lysine-specific demethylase 5B
4b5pB	−65.6825	Alpha-tubulin N-acetyltransferase 1
4gs4A	−65.3096	Alpha-tubulin N-acetyltransferase 1
5ij9B	−65.0098	Tubulin beta-3 chain
3znnA	−64.8223	D-amino-acid oxidase
5fyzA	−64.4186	Lysine-specific demethylase 5B
5fbeA	−63.8215	Complement factor D
4zhxE	−63.4517	5'-AMP-activated protein kinase subunit gamma-1
1c8tA	−63.2264	Stromelysin-1
4u7pA	−62.581	DNA (cytosine-5)-methyltransferase 3A

[1] Predicted knowledge-based docking scores with arbitrary units reflect relative binding free energies of a ligand to a protein.

Table S4. Potential human targets of troglitazone - stereoisomer d (Figure 1).

PDB ID with Chain	Docking Score [Arbitrary Units[1]]	Protein Name
4gs4A	−67.8725	Alpha-tubulin N-acetyltransferase 1
2ob0A	−67.3686	N-alpha-acetyltransferase 50
3uzxA	−67.027	3-oxo-5-beta-steroid 4-dehydrogenase
1fduA	−66.3763	Estradiol 17-beta-dehydrogenase 1
4b5pB	−66.3186	Alpha-tubulin N-acetyltransferase 1
3buvA	−65.5438	3-oxo-5-beta-steroid 4-dehydrogenase
4u7tA	−65.138	DNA (cytosine-5)-methyltransferase 3A
2wtvA	−64.4777	Aurora kinase A
2fs6A	−63.5546	Cellular retinoic acid-binding protein 2
1mncA	−63.5178	Neutrophil collagenase
5hh1A	−63.2317	N-alpha-acetyltransferase 60
5fyyA	−62.538	Lysine-specific demethylase 5B

[1] Predicted knowledge-based docking scores with arbitrary units reflect relative binding free energies of a ligand to a protein.

Table S5. Potential human targets of rosiglitazone - stereoisomer a (Figure 2).

PDB ID with Chain	Docking Score [Arbitrary Units[1]]	Protein Name
3f9zA	−63.3562	N-lysine methyltransferase KMT5A
4u7tA	−62.9032	DNA (cytosine-5)-methyltransferase 3A
2pd6A	−61.92	Estradiol 17-beta-dehydrogenase 8
1fduA	−61.8562	Estradiol 17-beta-dehydrogenase 1
4jijA	−61.7353	Matrix metalloproteinase-9
2pxxA	−59.9992	EEF1A lysine methyltransferase 4
5tdhA	−59.9391	Guanine nucleotide-binding protein G(i) subunit alpha-1
4f6uA	−58.9009	Cyclin-dependent kinase 8
1r9oA	−58.7018	Cytochrome P450 2C9

2bzgA	−58.596	Thiopurine S-methyltransferase

[1] Predicted knowledge-based docking scores with arbitrary units reflect relative binding free energies of a ligand to a protein.

Table S6. Potential human targets of rosiglitazone - stereoisomer b (Figure 2).

PDB ID with Chain	Docking Score [Arbitrary Units[1]]	Protein Name
1i3kA	−62.6382	UDP-glucose 4-epimerase
4u7pA	−61.8627	DNA (cytosine-5)-methyltransferase 3A
2zt5A	−61.6404	Glycine--tRNA ligase
4lk3A	−61.5583	UDP-glucuronic acid decarboxylase 1
4fvlA	−60.6104	Collagenase 3
4l27B	−59.7812	Cystathionine beta-synthase
4jijA	−59.3465	Matrix metalloproteinase-9
5cclA	−59.2019	Histone-lysine N-methyltransferase SMYD3
4lecA	−59.0789	Protein N-lysine methyltransferase METTL21A
4i8vA	−58.7122	Cytochrome P450 1A1
4af3A	−58.6548	Aurora kinase B

[1] Predicted knowledge-based docking scores with arbitrary units reflect relative binding free energies of a ligand to a protein.

Table S7. Potential target proteins of troglitazone in various organisms.

PDB ID with Chain	Docking score [Arbitrary Units[1]]	Organism	Protein Name
4kvxA	−69.5799	*Schizosaccharomyces pombe*	N-terminal acetyltransferase A complex catalytic subunit ard1
3uzxA	−68.1895	*Homo sapiens*	3-oxo-5-beta-steroid 4-dehydrogenase
5fyyA	−68.0396	*Homo sapiens*	Lysine-specific demethylase 5B
2wtvA	−66.1042	*Homo sapiens*	Aurora kinase A
5gy7A	−65.5891	*Escherichia coli*	UDP-glucose 4-epimerase
3v00C	−65.2407	*Bos taurus*	Guanine nucleotide-binding protein G(t) subunit alpha-1
4gs4A	−64.6551	*Homo sapiens*	Alpha-tubulin N-acetyltransferase 1
2chqA	−64.37	*Archaeoglobus fulgidus*	Replication factor C small subunit
1xhcA	−64.1425	*Pyrococcus furiosus*	NADH oxidase /nitrite reductase
1mncA	−64.0173	*Homo sapiens*	Neutrophil collagenase
5danA	−63.924	*Thermotoga maritima*	Oxidoreductase, aldo/keto reductase family
3buvA	−63.7212	*Homo sapiens*	3-oxo-5-beta-steroid 4-dehydrogenase

3fm1A	−63.6919	*Pleurotus eryngii*	Versatile peroxidase VPL2
1vp5A	−63.5643	*Thermotoga maritima*	Oxidoreductase, aldo/keto reductase family
5hh1A	−63.4527	*Homo sapiens*	N-alpha-acetyltransferase 60
1kpgA	−63.4338	*Mycobacterium tuberculosis*	Cyclopropane mycolic acid synthase 1
1zgdA	−63.2514	*Medicago sativa*	Chalcone reductase
5abqA	−63.088	*Pleurotus eryngii*	Versatile peroxidase VPL2
3znnA	−62.6779	*Homo sapiens*	D-amino-acid oxidase
5fyzA	−62.5991	*Homo sapiens*	Lysine-specific demethylase 5B
1c8TA	−62.3226	*Homo sapiens*	Stromelysin-1
4jn9A	−62.0995	*Chromobacterium violaceum*	DepH
1aj8A	−62.0631	*Pyrococcus furiosus*	Citrate synthase
1mn1A	−61.9114	*Phanerochaete chrysosporium*	Manganese peroxidase 1
4b5pB	−61.8538	*Homo sapiens*	Alpha-tubulin N-acetyltransferase 1
4wghA	−61.7674	*Klebsiella pneumoniae*	Aldehyde reductase
4fcnA	−61.6808	*Pleurotus eryngii*	Versatile peroxidase VPL2
3wb9A	−61.6706	*Symbiobacterium thermophilum*	Meso-diaminopimelate D-dehydrogenase
3s1sA	−61.4506	*Bacillus pumilus*	Restriction endonuclease BpuSI
3p4kA	−61.4362	*Mus musculus*	Mitogen-activated protein kinase 14
5dm2A	−61.3848	*Bacillus pumilus*	Methyltransferase domain family
3tmaA	−61.3608	*Thermus thermophilus*	tRNA (guanine(6)-N2)-methyltransferase
2jdcA	−61.2809	*Bacillus licheniformis*	Probable acetyltransferase
1a8pA	−61.1938	*Azotobacter vinelandii*	Ferredoxin--NADP reductase
4ntcA	−61.1912	*Aspergillus fumigatus*	Thioredoxin reductase gliT
5a89A	−61.1261	*Corynebacterium ammoniagenes*	Bifunctional riboflavin kinase/FMN adenylyltransferase

[1] Predicted knowledge-based docking scores with arbitrary units reflect relative binding free energies of a ligand to a protein.

Table S8. Potential target proteins of rosiglitazone in various organisms.

PDB ID with Chain	Docking Score [Arbitrary Units[1]]	Organism	Protein Name

2wfgA	−69.2009	*Candida albicans*	Potential cytosolic leucyl tRNA synthetase
5gy7A	−68.8871	*Escherichia coli*	UDP-glucose 4-epimerase
2gagB	−67.7125	*Stenotrophomonas maltophilia*	Heterotetrameric sarcosine oxidase beta-subunit
1ly8A	−63.9449	*Coprinopsis cinerea*	Peroxidase
1af7A	−63.0191	*Salmonella typhimurium*	Chemotaxis protein methyltransferase
3ad8B	−62.6366	*Corynebacterium sp, U-96*	Subunit beta of sarcosine oxidase
2uyhA	−62.0256	*Haemophilus haemolyticus*	Modification methylase HhaI
1ofwA	−62.0114	*Desulfovibrio desulfuricans*	Nine-heme cytochrome c
2ij3A	−61.9028	*Bacillus megaterium*	Bifunctional cytochrome P450/NADPH--P450 reductase
19hcA	−61.8681	*Desulfovibrio desulfuricans*	Nine-heme cytochrome c
4g5hA	−61.6155	*Staphylococcus aureus*	Capsular polysaccharide synthesis enzyme Cap8E
1kvsA	−61.3765	*Escherichia coli*	UDP-glucose 4-epimerase
1cclA	−61.0553	*Saccharomyces cerevisiae*	Cytochrome c peroxidase, mitochondrial
2c7rA	−60.9218	*Haemophilus haemolyticus*	Modification methylase HhaI
2yfpA	−60.584	*Aequorea victoria*	Green fluorescent protein
4jijA	−60.5409	*Homo sapiens*	Matrix metalloproteinase 9
1xkqA	−60.3102	*Caenorhabditis elegans*	Short-chain reductase family member (5D234)
5agiA	−60.0962	*Candida albicans*	Potential cytosolic leucyl tRNA synthetase
4lk3A	−59.8992	*Homo sapiens*	UDP-glucuronic acid decarboxylase 1
4c51A	−59.8991	*Mycobacterium tuberculosis*	Catalase-peroxidase
3f9zA	−59.8044	*Homo sapiens*	N-lysine methyltransferase KMT5A
7atjA	−59.7765	*Armoracia rusticana*	Peroxidase C1A
1jryA	−59.7231	*Shewanella frigidimarina*	Fumarate reductase flavoprotein subunit

6ccpA	−59.6452	*Saccharomyces cerevisiae*	Cytochrome c peroxidase, mitochondrial
1gw2A	−59.4391	*Armoracia rusticana*	Peroxidase C1A
1a9zA	−59.3416	*Escherichia coli*	UDP-glucose 4-epimerase
1cjtC	−59.2951	*Bos taurus*	Guanine nucleotide-binding protein G(s) subunit alpha isoforms short
1wy7A	−59.2852	*Pyrococcus horikoshii*	Uncharacterized protein
3b3rA	−59.018	*Streptomyces sp, SA-COO*	Cholesterol oxidase
4j56A	−58.9102	*Plasmodium falciparum*	Thioredoxin reductase 2
2vcsA	−58.7888	*Glycine max*	Cytosolic ascorbate peroxidase 1
1a8pA	−58.7845	*Azotobacter vinelandii*	Ferredoxin--NADP reductase
4u7pA	−58.767	*Homo sapiens*	DNA (cytosine-5)-methyltransferase 3A
4atjA	−58.6987	*Armoracia rusticana*	Peroxidase C1A
4fvlA	−58.6111	*Homo sapiens*	Collagenase 3
1cmuA	−58.6046	*Saccharomyces cerevisiae*	Cytochrome c peroxidase, mitochondrial
1sd0A	−58.5582	*Limulus polyphemus*	Arginine kinase
5agjA	−58.5393	*Candida albicans*	Potential cytosolic leucyl tRNA synthetase
3mq2A	−58.4694	*Streptoalloteichus tenebrarius*	16S rRNA methyltransferase
3v00C	−58.4579	*Bos taurus / Rattus norvegicus*	Guanine nucleotide-binding protein G(t) subunit alpha-1
1fduA	−58.4298	*Homo sapiens*	Estradiol 17-beta-dehydrogenase 1

[1] Predicted knowledge-based docking scores with arbitrary units reflect relative binding free energies of a ligand to a protein.
